# Adsorption of Rhodamine B dye from aqueous solution onto acid treated banana peel: Response surface methodology, kinetics and isotherm studies

**DOI:** 10.1371/journal.pone.0216878

**Published:** 2019-05-15

**Authors:** Adeleke Abdulrahman Oyekanmi, Akil Ahmad, Kaizar Hossain, Mohd Rafatullah

**Affiliations:** School of Industrial Technology, Universiti Sains Malaysia, Penang, Malaysia; Duke University Marine Laboratory, UNITED STATES

## Abstract

The adsorption of rhodamine B (RhB) using acid modified banana peels has been examined. Chemical characteristics of the adsorbents were observed in order to determine active functional groups. The major functional groups on the surface were OH, C = O, C = C and C-O-C. Interactions between operational parameters were studied using the central composite design (CCD) of response surface methodology (RSM). The predictions of the model output indicated that operational factors influenced responses at a confidence level of 95% (P<0.05). The optimum conditions for adsorption were pH 2 at a 0.2 g/L dose within 60 minutes of contact time. Isotherm studies were carried out using the optimized process variables. The data revealed that RhB adsorption fitted the Langmuir isotherm equation while the reduction of COD followed the Freundlich isotherm. Kinetic experiments fitted the pseudo second order model for RhB removal and COD reduction. The adsorption mechanism was not the only rate controlling step. Diffusion through the boundary layer described the pattern of adsorption.

## Introduction

Effluents generated from rubber, plastic, leather and textile processing plants contain different kinds of synthetic dyes, such as malachite green, methyl violet, azure dye, indigo carmine and Rhodamine B (RhB) [1]. The discharge of these dyes into the receiving waters produce negative biological and ecological effects, which have the propensity of inhibiting aquatic growth and consequently resulting in the death of aquatic species. The presence of recalcitrant pollutants such as dyes in the receiving water affects the penetration of sunlight, resulting in anoxic conditions that have a profound effect on photosynthetic activities and aquatic respiration. The discharge can also be lethal to the environment. Synthetic dyes are complex in nature due to their aromatic structures, which are nonbiodegradable as a result of their optical, thermal and physic chemical stability [2, 3]. These compounds are hazardous and can be carcinogenic [4]. Considering the implication of the effect of the discharge into the water bodies and the environment, stringent measures and regulations, including many treatment methods, have been explored as a measure of protecting aquatic life and the ecosystem.

However, due to the complexity of the structure and thermal stability of synthetic dyes in water, biological methods of treatment have been less successful [5]. In recent years, several physico-chemical methods have been reported for the treatment of wastewater containing dye, including forward and reverse osmosis [6, 7], coagulation [8], coagulation-flocculation [9], Fenton, Photo–Fenton and solar Fenton reactions [10, 11], electrocoagulation [12], chemical oxidation [13], solvent extraction [14], ion exchange [15] and adsorption [16]. Among the conventional methods, adsorption is a widely applicable and superior technique owing to the low cost of treatment and ease of design and operation; the method has rapid kinetics and is very effective for the treatment of effluents [17, 18]. The efficacy of adsorption is determined by the ability of adsorbents to remove priority pollutants from the effluent [19, 20]. Some of the natural adsorbents used for dye removal are economical and abundant in nature, such as Prunus Dulcis [21], mangopeel [22], rambutan seed [23], litchi pericarps [24], coconut shell [25], cupuassu shell [26] and jackfruit [27]. There are no studies available in the literature describing the adsorption and optimization of operational variables for the removal of RhB using banana peel. Various functional groups, such as–COOH, -OH, -C = O and amines, present on the surface of the adsorbent have the potential of providing active binding sites for the attachment of adsorbate from solution. To improve on the performance of the adsorbent, surface modification and functionalization can improve the adsorption capacity [28].

In addition, optimization of operational conditions of the adsorption process can improve the reduction in the response parameters in the adsorbate. Consequently, the scope of this study is not limited to the removal efficiency of the modified banana peel but also examines the effect of the interaction of operational conditions for the removal of RhB and COD reduction in test solutions. The multi component optimization tool response surface methodology (RSM) is a very comprehensive, reliable and time saving technique to achieve this purpose when compared to the conventional optimization process. This process is not very effective for optimization because it involves fixing a parameter in terms of other parameters. Similarly, the application of conventional techniques for the optimization of process variables cannot determine the effect of the interaction of parameters in the adsorption process [29]. RSM is a statistical tool that is very effective for designing, analyzing and optimizing the effect of independent factors for the prediction of response output. The uniqueness of RSM is due to its ability to optimize multicomponent independent factors with precision in order to achieve better adsorption performance [30]. The central composite design (CCD) of the RSM is widely used for design and optimization purposes to achieve better optimization output.

The main objective of the present work was to observe the effect of the operational conditions on the efficiency of the modified banana peel on the adsorption of RhB and COD reduction using the CCD of the RSM. The regression model of the CCD was used for the analysis of the interaction of independent factors. The statistical analysis and significance of the interaction of the process variables was interpreted using a three dimensional response surface. Equilibrium data from the batch study at the optimized condition were fitted to the isotherm. Furthermore, kinetic equations were used to observe the mechanism of the adsorption.

## Materials and methods

### Adsorbent

The batch study for the adsorption of RhB and COD reduction was executed using acid treated banana peels. The material used for the study was collected from the local market in Restu, Penang, Malaysia. The transportation, processing and storage were accomplished according to international standard methods [31]. The peels were thoroughly washed with tap water to eliminate dirt and surface impurities. Next, the peels were chopped into desirable sizes of approximately (1.0–2.0 cm). The chopped materials were oven dried at 105°C for 24 hrs. The dried peels were washed again with water at 100 °C and oven dried at 105 °C for 24 hrs, after which the dried peels were ground using a Retsch Mill Grinder to obtain a working particle size in the range of 150 to 250 μm. A mild acid (Phosphoric acid-H_3_PO_4_) was used for surface modification of adsorbents in the study. For this modification, 100 g of powder banana peel was soaked in 1 L of 0.5 N H_3_PO_4_. The ratio of 1 g powder: 20 mL of H_3_PO_4_ was applied for 4 hrs [32]. After that, the treated banana peel was washed with demineralized water. The material was dried in an oven at 105°C for 24 hrs. Potassium permanganate (KMnO_4_) was applied to further oxidize the adsorbent surface. For this step, 100 g of treated banana powder was soaked in 1 L of 0.1 N KMnO_4_ for 24 hours [33], after which the sample was washed using demineralized water until the pH became neutral. After that, the pretreated banana peel adsorbent was oven dried at 80°C for 24 hrs and kept in airtight plastic bottles labeled according to the sizes.

### Characterization of adsorbent

The morphology of the raw and modified banana peel was obtained from the scanning electron microscopy micrographs (ZEISS, Germany). The sample was prepared on an aluminum plate. Prior to analysis, samples were wrapped with gold foil using a sputter coater (SCD050) to improve the conductivity. The morphology of the adsorbent was captured at an accelerating voltage of 15 kV. The surface characteristics of the adsorbents were described on the basis of the active available functional groups. The active sites on the adsorbent surface were obtained from Fourier Transform Irradiation (FT-IR) using the transmittance mode conducted over wavelengths of 500 and 4000 cm^-1^. The peaks were obtained in less than 30 seconds of scanning time.

### Adsorbate

**Fig 1:**
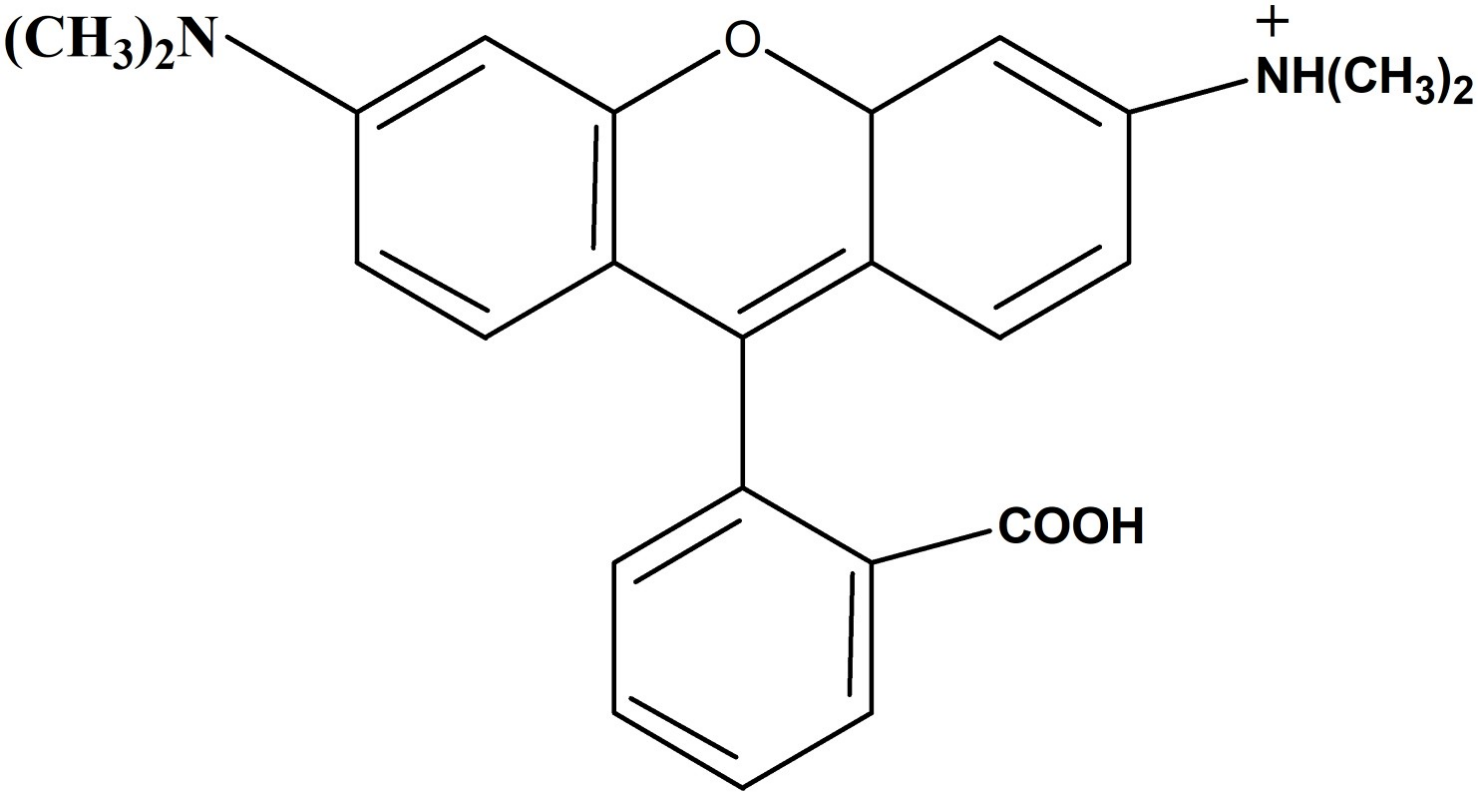
Chemical structure of Rhodamine B.

The synthetic dye (Rhodamine B) (RhB) used in this study was supplied by R & M in powdered form. It has a chemical structure of C_28_H_31_CIN_2_O_3_ (Fig 1). The molecular weight of RhB was observed as 479.02 g/mol and the maximum wavelength (*λ*_*max*_) was obtained at 543 nm. A standard solution (1000 mg/L) was prepared by dissolving 1 g of RhB powder into 1 L of demineralized water in a volumetric flask. The batch adsorption study was performed for the removal of RhB and COD reduction from the aqueous solution.

### Experimental design

A factorial design (2^3^) was used in this study to determine the linear effect and interaction of three (3) independent process factors. The factors were assigned *x*_1_, *x*_2_, *x*_3_, representing pH, adsorbent dosage and contact time, respectively, for the adsorption of RhB and COD in aqueous medium by response surface methodology. *Y*_1_ and *Y*_2_ represent color and COD, respectively. A total of 20 experiments including 7 centered points were investigated using the central composite design expert (6.0.10) method. The batch study was conducted in triplicate and recorded in averages to reduce the variability in the results. The optimization of the variable factors was determined in terms of response factors according to Eq 1:

$$Y=f(x_{1},\;x_{2},\;x_{3}\ldots \ldots x_{n})+ƹ$$ (1)

where *Y* illustrates the response parameter, *f* denotes the function of response parameter, ƹ signifies experimental error and (*x*_1_, *x*_2_, *x*_3_ … … *x*_*n*_) are the independent process factors.

The expected response is denoted by Eq 2:

$$E(y)=f(x_{1},\;x_{2},\;x_{3},\;x_{4},\;x_{5},x_{6})=ŋ$$ (2)

The evaluation of the surface area is expressed as Eq 3:

$$ŋ=f(x_{1},\;x_{2},\;x_{3},\;x_{4},\;x_{5},x_{6})$$ (3)

The second-order regression coefficient for the adsorption of RhB and COD is determined according to Eq 4:

$$Y=\beta 0+\sum_{i=1}^{k}\beta_{i}x_{i}+\sum_{i=1}^{k}\beta_{ii}x_{i}^{2}+\sum_{i=1}^{n}\sum_{i<j}^{n}\beta_{ij}x_{i}x_{j}$$ (4)

where *Y indicates the expected response for the adsorption efficiency*, *β*_0_, *β*_*i*_, *β*_*ii*_, *and β*_*ij*_ *denotes the regression coefficients*, *x*_*i*_ *represents the coded variables*, *and x*_*j*_ *illustrates the process variables in coded form*, *while k is an expression of the number of process variables*.

The natural (*ε*_*i*_) and the coded variables (*x*_*i*_) are evaluated according to Eq 5:

$$x_{i}=\frac{\varepsilon_{i}-[HL+LL]/2}{[HL-LL]/2}$$ (5)

*where x*_*i*_ *represents maximum HL (+1)*, *minimum LL (-1) and center points (0) of the coded variable*, *and ε*_*i*_ *illustrates a natural variable (*Table 1*)*.

**Table 1:** The coded and un-coded levels of the independent variables.

Factor	Symbol	Level		
		Low (-1)	Middle (0)	High (+1)
**pH**	*x*_1_	2	6	10
**Adsorbent dosage (g L**^**-1**^**)**	*x*_2_	0.2	0.6	1
**Contact time (h)**	*x*_3_	60	150	240

The analysis of the first order of the model was used to generate a response surface equation using expert design software. This was to determine the effect and significance of the interaction of the operational factors on the response output. The prediction of the interaction of process variables was achieved using analysis of variance (ANOVA, p<0.05), which is equivalent to the 95% confidence level for the determination of the linear fit. The three dimensional surface of the RSM was used for the analysis of the interaction between the independent factors for the removal of RhB and COD reduction.

### Batch adsorption studies

Equilibrium experiments were performed using the optimum working parameters (Table 1) for the removal of RhB and COD reduction. The operational conditions were 2–10, 0.2–1 g/L, and 0.5–3.0 h, representing pH, adsorbent dosage and contact time, respectively, for the 20 experimental analyses in 250 mL Erlenmeyer conical flasks. Initial dye concentrations between 20–100 mg/L were prepared in 50 mL volumetric flasks. The equilibrium experiments were performed in an orbital shaker at 150 rpm. The pH adjustment was achieved using 0.1 M sodium hydroxide (NaOH) and 0.1 M hydrochloric acid (HCl) to obtain the required pH condition of the analyte. The aqueous solution was obtained from the supernatant at equilibrium contact time using a 0.45 μm filter membrane, after which the concentrations were determined. The concentration of COD was analyzed using the closed reflux method according to the guidelines described in No. 5220 A standard method [34]. The initial concentration of RhB was obtained at 543 nm (λmax) using an Elico–UV spectrophotometer model (SL159). The supernatant was filtered at equilibrium and analyzed at 543 nm from the calibration curve to obtain the amount of RhB adsorbed [35].

The amount of RhB and COD adsorbed at equilibrium concentration was evaluated according to Eq 6:

$$qe=(\frac{Co-Ce}{Co})\times 100\%$$ (6)

where *is the amount of RhB adsorbed; Co* and Ce *represent initial liquid phase and final concentrations of RhB and COD*, *respectively*.

### Adsorption isotherm studies

An equilibrium isotherm was used to illustrate the removal efficiency of the banana peel and was evaluated from the expression (*qe* versus Ce). Equilibrium data for batch adsorption were fitted to two commonly used isotherms, the Langmuir and the Freundlich. The Langmuir isotherm validates the hypothesis that the adsorption of molecules of the adsorbate on the surface has equal activation energy on a monolayer surface.

An expression to describe the Langmuir equation is expressed by Eq 7:

$$q_{e}=\frac{q_{maxK_{L\;C_{e}}}}{1+K_{L\;C_{e}}}$$ (7)

Where

*C*_*e*_ illustrates amount of RhB adsorption at final concentration (mg/L);

*q*_*max*_ describes monolayer adsorption efficiency of the adsorbent (mg/g); and

*k*_*L*_ signifies the adsorption constant (L/g).

The linearized plot of $\frac{1}{q_{e}}$ versus $\frac{1}{C_{e}}$ is represented by the slope *q*_*mon*_ and intercept *K*_*L*_ obtained from the graph. The Langmuir isotherm is defined in relation to the dimensionless constant known as the separation factor. This is expressed according to Eq 8. The *q*_*max*_ is defined as the amount of adsorption of RhB that occurred on the monolayer surface.

$$R_{L\;}=\frac{1}{1+K_{L}C_{O}}$$ (8)

where *C*_0_ determines the initial RhB concentration (mg/L) and *K*_*L*_ describes the shape of the isotherm. Adsorption is irreversible if (*R*_*L*_ = 0), linear if (*R*_*L*_ = 1), favourable if (0<*R*_*L*_<1), and unfavourable if (*R*_*L*_ >1). The Freundlich isotherm equation is given as Eq 9.

$$q_{e}=K_{f}.{C_{e}}^{\frac{1}{n}}$$ (9)

A linearized plot of log *q*_*e*_ versus log *C*_e_ illustrates the Freundlich equation to describe the surface heterogeneity of the adsorbent. The constants n and *K*_*f*_ represent the slope and intercept of the graph, respectively. *K*_*f*_ is the coefficient that defines the adsorption capacity, while n describes the adsorption intensity, which explains the favorability of the adsorption. A coefficient of $\frac{1}{n}$ < 1 indicates favourable adsorption while $\frac{1}{n}$ > 1 describes a nonfavourable adsorption on a heterogeneous surface.

### Adsorption kinetic studies

The adsorption of analyte on the adsorbent surface is time-dependent. Adsorption kinetics are governed by mass transfer and reactions on the surface of the adsorption site within the adsorbent particles from the solution. Kinetics of adsorption could occur either by external or intraparticle diffusion. In the case of the adsorption equilibrium of porous surfaces, the mass transfer of the solute to the site of adsorption is usually inhibited by mass transfer resistance. This determines the required time to achieve equilibrium. The kinetics of adsorption are determined by the rate of diffusion within the adsorbent particle and towards the external surface. The evaluation of RhB removal is necessary to obtain the optimum conditions for the full-scale batch study. The kinetics study of RhB removal at the equilibrium contact time was investigated using widely applied kinetic models. All the kinetic parameters (pseudo first order, pseudo second order, Elovich and Intraparticle) are represented by the following equations:

$$\frac{dq_{t}}{dt}=k_{1}(q_{e}-q_{t})$$ (10)

$$\frac{dq_{t}}{dt}=k_{2}(q_{e}-q_{t}{)}^{2}$$ (11)

$$q_{t\;}=\frac{1}{\beta }\;(ln\;\alpha \beta )+\frac{1}{\beta }ln\;t$$ (12)

$$q_{t\;}=k_{i}t^{0.5}+C$$ (13)

where *q*_*e*_ and *q*_*t*_ illustrates the amount of RhB adsorbed at equilibrium contact time (t), *k*_1_ determines the rate constant of pseudo-first order ($\frac{1}{min}$), *K*_2_ is the rate constant of pseudo-first order adsorption, *α* defines the initial rate of adsorption (mggmin), *β* is the desorption constant, *h*_1_ is the initial rate of adsorption (mg/g-min) of pseudo first order, and *h*_2_ is the initial rate of adsorption of pseudo second order (mg/g-min).

The pseudo-first order model expresses the theoretical hypothesis that the rate of adsorption of RhB and the difference between the amounts of dye adsorbed at a specific time with the amount of RhB adsorbed is proportional at equilibrium concentration. The pseudo second order model gives the assumption that the adsorption kinetics of the dye is proportional to the square of the difference between the amounts of RhB adsorbed and COD reduction at equilibrium at a specified time. The Elovich model assumes that different levels of activation energy occur during stages of adsorption between the adsorbent heterogeneous surface and the site of adsorption. An intraparticle diffusion-based model was adopted to examine the role of diffusion in the adsorption process of RhB and COD on the acid modified banana peel. In this study, the diffusion controlled mechanism was considered appropriate to validate the pattern of adsorption [25]. The intraparticle diffusion rate equation is denoted by Eq 13.

## Results and discussion

### Characterization of banana peel

**Fig 2:**
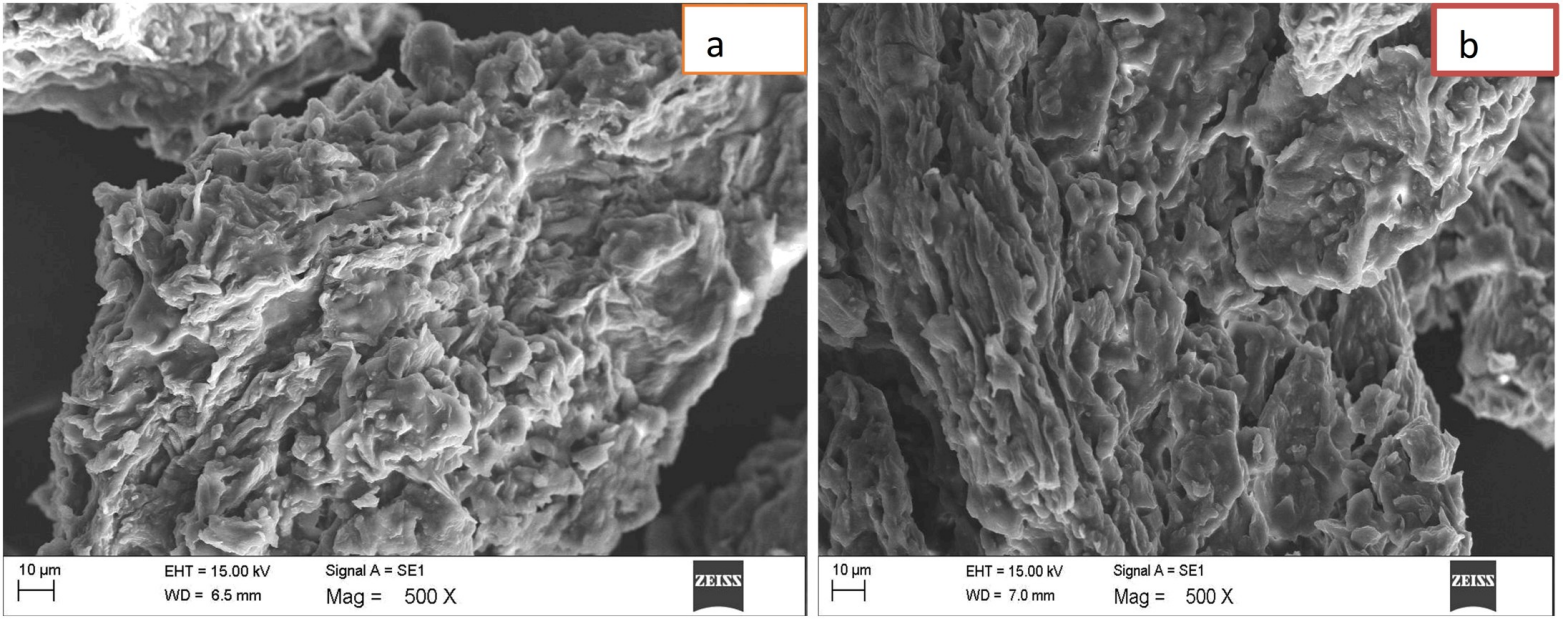
SEM images (a) Raw banana peel (b) Acid modified banana peel.

**Fig 3:**
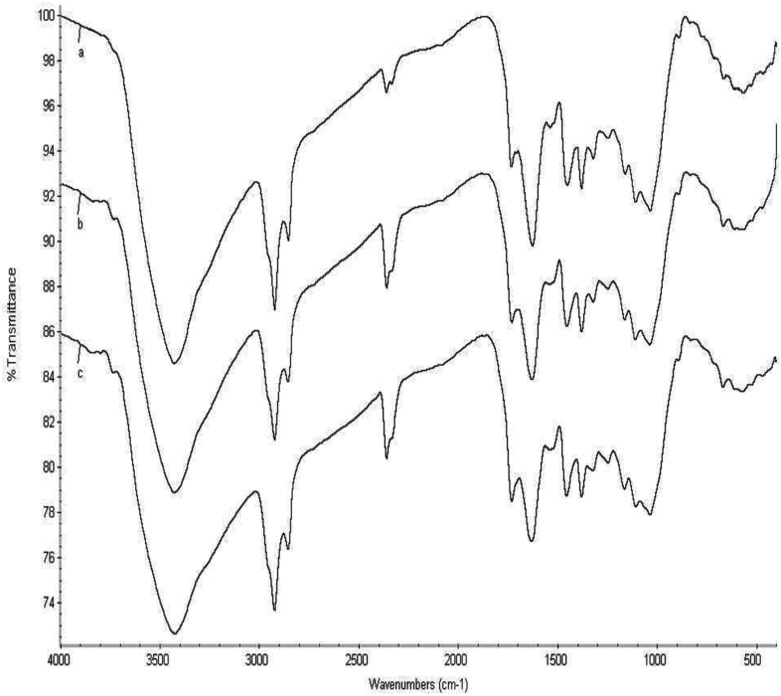
FT-IR spectrum for a) raw banana peel b) acid modified banana peel c) banana peel after adsorption of RhB dye.

The SEM micrograph of the unmodified and modified banana peel was obtained at a resolution of x500 magnification using a particle size of 10 μm. The surface morphology of the raw banana peel (Fig 2a) was observed to be slightly different from the modified banana peel (Fig 2b). In Fig 2a, a rough surface of the morphology of the raw banana peel was revealed through the micrograph, and particles were tightly packed with no obvious open porous surface. This is owing to the presence of pectin, lignin and viscous compounds [36]. The modified banana peel can be observed to have open pores with a rough and irregular surface as a result of the chemical modification of the surface. Due to the modification of the surface, oxidation of lignin occurred, thereby generating hydroxyl, carbonyl and carboxyl, which enhance the solubility of lignin in alkaline solution [37]. The available active functional group was used to determine the bonding and cross-linkage on the surface of the adsorbents. This was obtained using the FT-IR spectra. The FT-IR spectra of banana peel appeared similar to the observations of Memon et al. 2008 [38]. Fig 3a indicated that the major bands of 3425.63, 2924.68 and 1732.37 were assigned to the OH, C-H stretching vibration, and C = O stretching vibration in carboxylic acid, respectively, and C-H bending vibrations in CH_2_ and CH_3_, C-O-C vibrations in ether and C-O-H stretching vibrations in secondary cyclic alcohol, respectively (Fig 3b). There was a shift in CH stretching vibration as a result of increased intensity at spectra 2855.40–2856.48, indicating the presence of the methoxyl group as a result of the removal of lipids and lignin. There was a noticeable band at 1628.30, which is attributed to the presence of C = C stretching vibration. The OH and carboxylic groups strongly influenced the adsorption of RhB. A reduction in the peak of C-O-H stretching vibration in secondary cyclic alcohols was observed in Fig 3b. The observation indicated the significance of the available oxygen in the hydroxyl and carbonyl groups, which has a profound effect on the surface characteristics of the banana peel. This was observed through the dispersion in aqueous solution forming a stable suspension that influences the adsorption of RhB [39,40]. A perceptible alteration up to 22–9 cm^-1^ has been observed in Fig 3c in–OH and–COOH groups. It confirms that these functional groups are responsible for binding the RhB dye through electrostatic/H-bonding interactions [41].

### Screening of independent process variables

A batch adsorption study was undertaken to examine the interaction of the operational parameters for RhB removal and COD reduction on modified banana peels. A central composite design (CCD) was applied to determine the effect of the influential factors on the adsorption capacity. A total of twenty (20) experimental runs consisting of seven (7) central points were optimized using three influential parameters of independent variables for the removal of RhB and COD reduction as response factors from aqueous solution. This was to determine the effect of the interaction of operational factors that improved the adsorption capacity of modified banana peels (S1 Appendix). The results showed that the operational process variables influenced the reduction in the responses as a result of improved characteristics of the surface. The effectiveness of the modified surface is attributed to the modification of the surface that generated cross linkages of the active functional groups of the surface. The optimization study was conducted to determine the optimum operational process variables that influenced the adsorption of response factors and the effect of the interaction of the variables in the adsorption process. This was determined using analysis of variance (ANOVA, P<0.05). The percentage removal of the RhB and COD reduction was evaluated according to Eq 6. Screening was obtained in a batch experiment to determine the effect of the variable factors of pH (2–10), adsorbent dosage (0.2–1.0 g) and contact time (1–4 h). The regression coefficients of the linear and quadratic equations and the interaction between the operational factors in the model was examined using the response surface methodology. The effect of interaction of process variables can be synergistic, antagonistic or reciprocal [42]. The effect of the operational factors was considered as significant at a confidence level of 95% (P< 0.05). The regression coefficient for the linear and quadratic equations and the interaction between variable factors is presented in Table 2. The overall prediction of the model output in terms of the operational factors indicated that the model suitably predicted the adsorption of RhB and COD reduction (P<0.01).

**Table 2:** Regression coefficient for the adsorption of RhB and COD reduction and the significance of linear quadratic model.

Term	Coefficient		Standard Error		F-Value		P-Value	
	RhB	COD	RhB	COD	RhB	COD	RhB	COD
**Model**	**5778.78**	**2408.66**	**32.9**	**3.24**	**5.33**	**3.67**	**0.0076**	**0.0274**
**X**_**1**_	-17.56	-8.90	-17.56	2.31	34.92	14.84	0.0001	0.0032
**X**_**2**_	2.64	2.94	2.64	2.31	0.79	1.62	0.3950	0.2317
**X**_**3**_	-6.50	-0.18	-6.50	2.31	4.79	0.0057	0.0536	0.9410
**X**_**1**_^**2**^	3.10	-2.87	3.10	2.25	1.15	1.63	0.3089	0.2306
**X**_**2**_^**2**^	1.55	6.39	1.55	2.25	0.29	8.07	0.6046	0.0175
**X**_**3**_^**3**^	2.13	4.24	2.13	2.25	0.54	3.56	0.4784	0.0884
**X**_**1**_**X**_**2**_	-6.13	-2.77	-6.13	3.02	2.50	0.84	0.1451	0.3801
**X**_**1**_**X**_**3**_	6.61	-2.58	6.61	3.02	2.90	0.73	0.1194	0.4124
**X**_**2**_**X**_**3**_	-2.32	3.56	-2.32	3.02	0.36	1.39	0.5633	0.2649

The results obtained for the process variables demonstrated the significance of the effect of the negative coefficient of pH, which implies that increases in pH may likely reduce the effectiveness of the banana peel for the removal of RhB and COD reduction. However, there was no significance as a result of the negative coefficient of contact time that would lead to an increase in the adsorption of RhB and COD reduction. Meanwhile, the linear effect of dosage was observed to be nonsignificant to the reduction of responses. It was observed that the quadratic coefficient of pH reduced the effectiveness of the adsorption process of RhB but the negative effect of the coefficient would increase the adsorption capacity of banana peel for the reduction of COD. In addition, the quadratic coefficient of dosage inhibited the process of adsorption of RhB but improved the reduction of COD. The regression model was used to examine the effect of the interaction of the process variables. The effect of the negative coefficient of interaction of pH and dosage was synergistic, which led to improved performance of the adsorbent for the adsorption of the responses, while the effect of the negative coefficients of contact time and pH improved the reduction of COD. On the other hand, both operational factors were observed to be antagonistic to the removal of RhB. However, in the case of the interaction of dosage and contact time, the effect of the negative coefficient was synergistic, which implies that there was increase in the adsorption of RhB. In addition, the coefficient of the interaction of dosage and contact time reduced the adsorption capacity of the modified banana peel for the reduction of COD.

The significance of the regression coefficient for the reduction of the responses on the modified banana peel was evaluated using the second-order polynomial model. The result is presented in coded factors of the process variables in Eqs 14 and 15.

$$Y_{1}=+32.90-17.56x_{1}+2.64x_{2}-6.50x_{3}+3.10x_{1}+1.5x_{2}+2.13x_{3}-6.13x_{1}x_{2}+6.61x_{1}x_{3}-2.3x_{2}x_{3}$$ (14)

$$Y_{2}=+52.72-8.90x_{1}+2.94x_{2}-0.18x_{3}-2.87x_{1}+6.39x_{2}+4.24x_{3}-2.77x_{1}x_{2}-2.58x_{1}x_{3}+3.56x_{2}x_{3}$$ (15)

where *Y*_1_ represents the removal percentage of RhB on acid treated banana peel and *Y*_2_ is the reduction percentage of COD on acid treated banana peel.

The coefficient of the regression obtained from the regression equation is expressed in the S1 Appendix. There was agreement between the expected and the experimental values. The analysis of variance (ANOVA) for the quadratic model is shown in Table 3. It was observed that the model was suitable for the prediction of the adsorption of RhB and COD reduction with a statistical significance of P < 0.05 at a confidence level of 95%. The lack of fit for the model was not substantial (P > 0.05), which indicated the suitability of the model for the analysis of the adsorption process within the range of the investigated process variable. The analysis of variance indicated a strong agreement between the investigated data and the mathematical model.

**Table 3:** Analysis of variance (ANOVA) of the quadratic model for removal of RhB and COD reduction onto banana peel.

Source	Degree of freedom	Sum of squares		Mean square		F value		P value	
		RhB^a^	COD^b^	RhB	COD	RhB	COD	RhB	COD
**Model**	9	5778.78	2408.66	642.09	267.63	5.33	3.67	0.0076	0.0274
**Residual error**	10	1205.42	728.85	120.54	72.85	34.92			
**Lack-of-fit**	5	622.85	176.33	124.57	35.27	0.79	1.07	0.4717	0.8821
**Pure error**	5	582.57	552.17	116.51	110.43	4.79			
**Total**	19	6984.20	604.95						

^a^R^2^ = 82.74%, R^2^ (adj) = 67.21%

^b^R^2^ = 76.78%; R^2^ (adj) = 55.88%%

**Fig 4:**
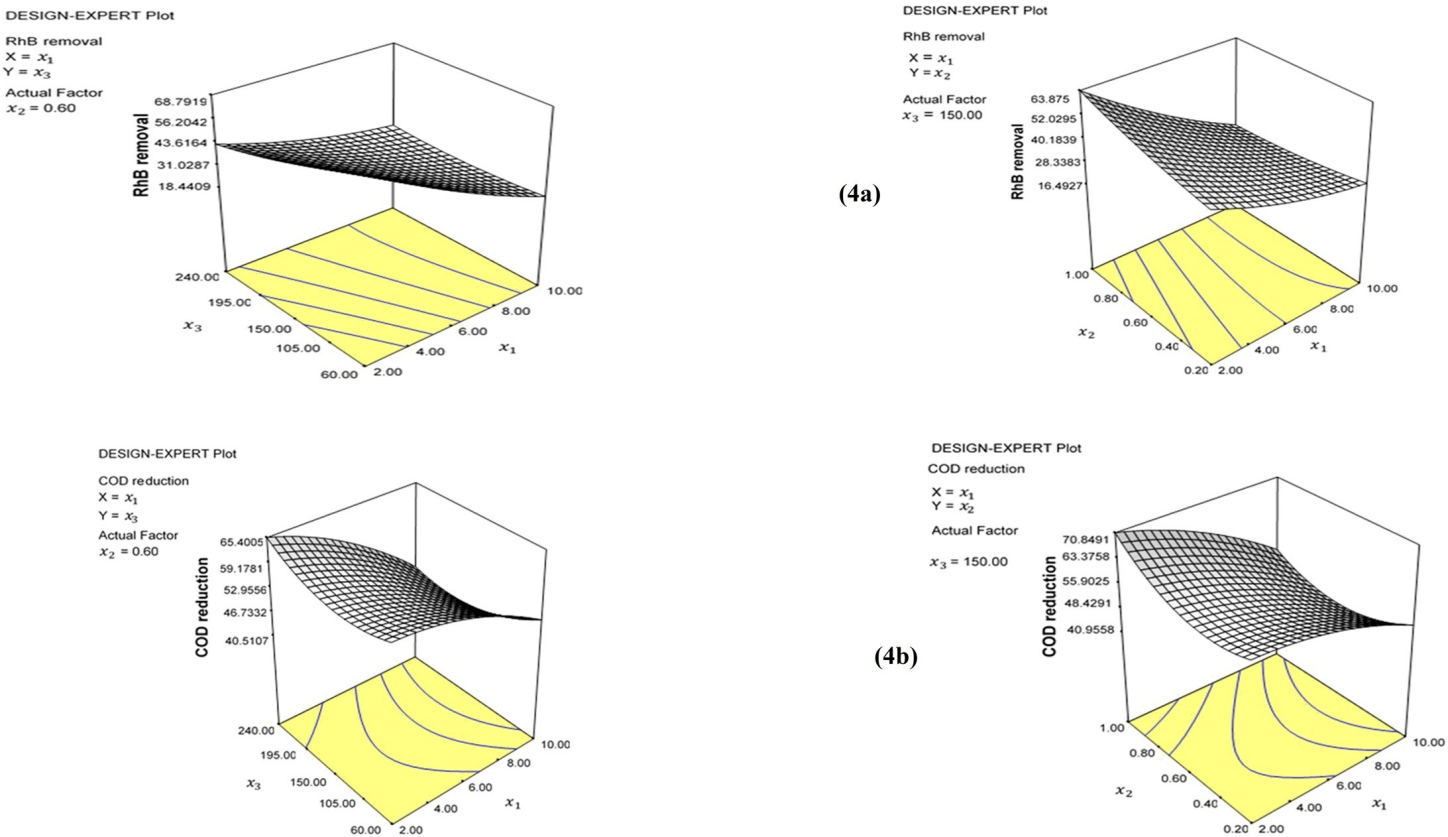
Three dimensional response surface for illustrating the adsorption of a) RhB removal onto banana peel b) COD reduction onto banana peel.

The effect of the synergy between the process variable was obtained at the center of the investigated independent parameters using the range of the actual factor. The level of the interaction of the parameters was obtained using RSM. A significant statistical interaction was achieved between contact time and pH for RhB removal by 68.79%. Similarly, the removal of RhB by 63.88% was achieved based on the interaction of pH and adsorbent dosage (Fig 4a). The interaction between pH and adsorbent dosage resulted in the reduction of COD by 70.85%, while the interaction between pH and contact time reduced the concentration of COD by 67.38% (Fig 4b).

### Optimization of the adsorption process

**Fig 5:**
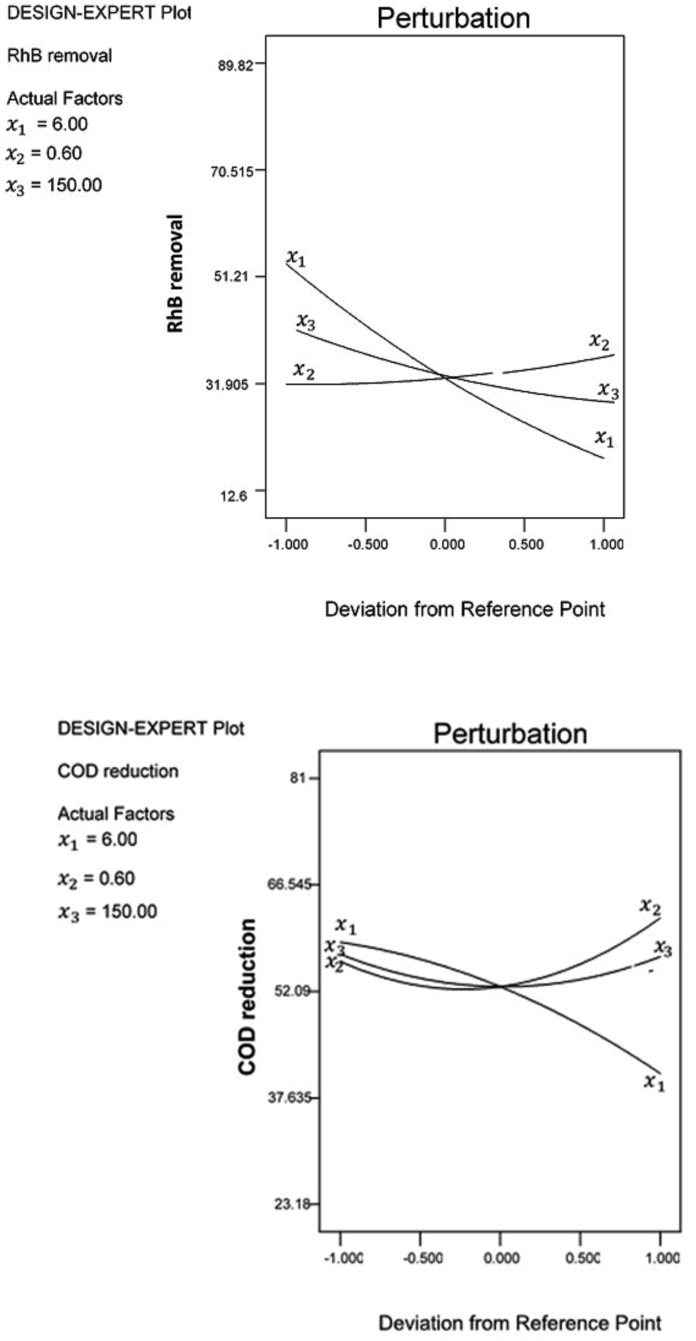
Interaction between process variables (a) RhB removal (b) COD reduction.

The interaction between working variables using conventional methods is difficult. Hence, the synergistic effect and predictions of operational variables are usually based on assumptions. The ability to investigate operational variables concurrently and to evaluate the extent of interaction can be better achieved using RSM. The optimization of working variables for the adsorption of RhB and COD reduction onto acid modified banana peel was investigated using the response surface. The optimized conditions for the adsorption of RhB and COD reduction was obtained from the result of the screening of the operational factors (S1 Appendix). The optimum condition of process factors was obtained at pH 2 and 0.2 g/L adsorbent dosage within 60 minutes contact time. Under this condition, there was a strong interaction of (P<0.05) from the coefficient of regression of the quadratic model. The actual and predicted values obtained for the adsorption of RhB and COD reduction were 89.82 vs. 81.43% and 69.96 vs. 69.12%, respectively. There was strong interaction between process variables for the reduction of responses at a confidence level of 95% (P < 0.05) (Fig 5).

### Adsorption isotherm

**Fig 6:**
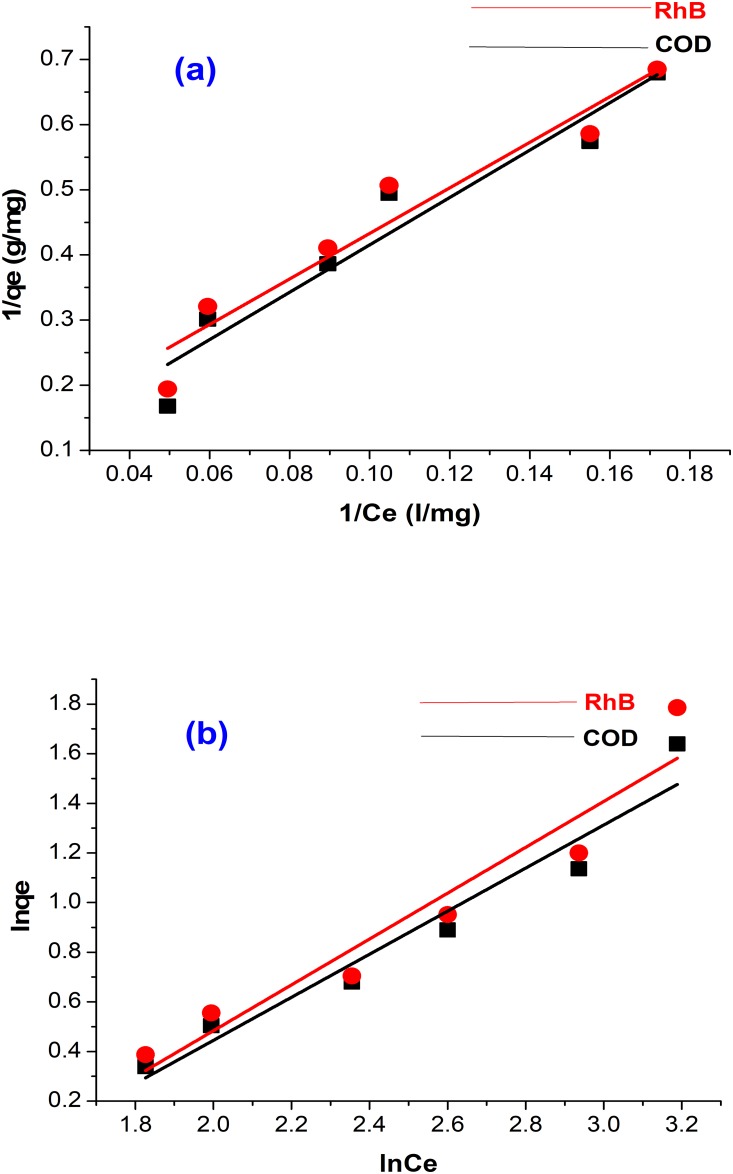
Adsorption isotherms for RhB and COD by acid modified banana peel at 28 °C (a) Langmuir isotherms for RhB and COD (b) Freundlich isotherms for RhB and COD.

The suitability of isotherm equations for the prediction of the pattern of adsorption is very important for industrial design and application. Equilibrium adsorption data for the uptake of RhB and COD reduction by acid modified banana peel were studied using the Langmuir and Freundlich models. A linearized form of Langmuir equation for RhB and COD is illustrated in Fig 6a and the Freundlich equation is represented in Fig 6b. The regression coefficients of the Langmuir and Freundlich equations are given in Table 4. The equilibrium data were observed to fit better to the Langmuir isotherm equations with a correlation coefficient (R^2^) of 0.9323 compared to Freundlich (0.9213). The values showed that the adsorption of RhB followed the Langmuir equation, which is an indication that the adsorption sites and the energies of acid modified banana peel were homogeneously distributed. From the expression of Eq 8, the dimensionless equilibrium separation factor indicated that a pattern of 0<*R*_*L*_< 1 was followed, which indicated favourable adsorption of RhB on acid modified banana peel. The adsorption capacity (*q*_*m*_) of the adsorbent (9.5220 *mg*/*g*) was observed to be higher than the adsorption of RhB onto banana pith (8.5 *mg*/*g*) [43], carbon nanosphere (1.15 *mg*/*g*) [44], and Tamarind fruit shell carbon (3.94 *mg*/*g*). On the other hand, the Freundlich isotherm fitted better for the reduction of COD, with an R^2^ value of 0.9642 compared to the Langmuir isotherm (0.9548). This suggests that the adsorption process was not on uniform sites but the uptake capacity of the adsorbent was on a heterogeneous surface. The observed pattern of adsorption was similar to the study of Inyinbor et al. [45], where it was observed that RhB removal was more similar to the Freundlich isotherm model.

**Table 4:** Langmuir and Freundlich constants for the adsorption of RhB and COD reduction onto acid modified banana peel.

Parameter	Acid modified banana peel						
	Langmuir Isotherm				Freundlich Isotherm		
	$q_{max}\;(\frac{mg}{g})$	$K_{L}\;(\frac{l}{mg})$	*R*_*L*_	*R*^2^	*K*_*F*_	n	*R*^2^
RhB	9.5220	0.3884	0.0489	0.9323	0.2992	1.1924	0.9213
COD	9.8338	0.3847	0.0494	0.9548	0.3171	1.2359	0.9642

### Adsorption kinetics

**Fig 7:**
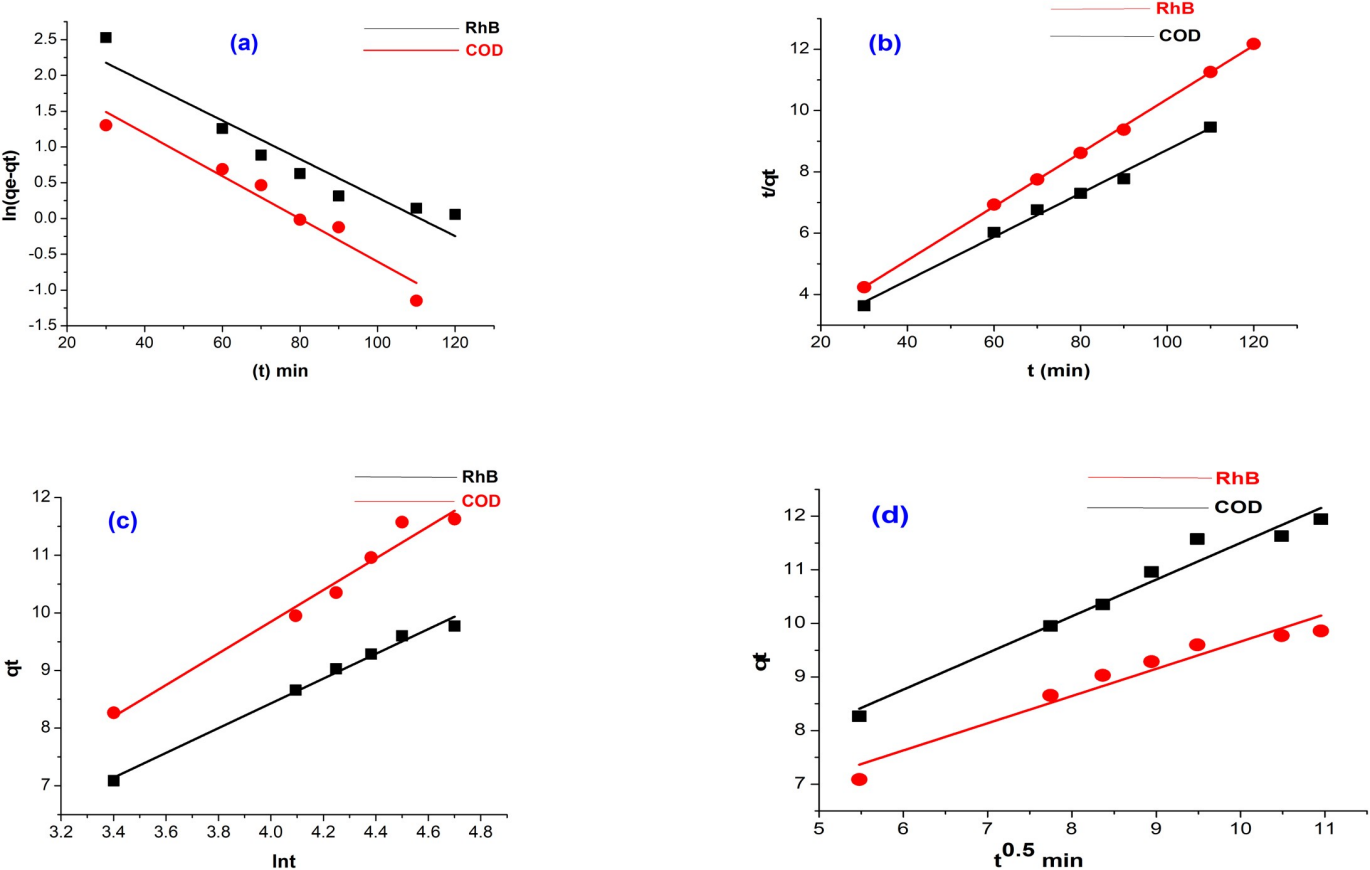
Kinetic studies for RhB adsorption and COD reduction onto acid modified banana peel (a) Pseudo first order kinetics for RhB and COD (b) Pseudo second order kinetics for RhB and COD (c) Elovich kinetic study for RhB and COD (d) Intraparticle transport for RhB and COD (Experimental conditions: Adsorbent mass 3.5 g/ 50 ml, temperature = 28 °C, pH 2, agitation speed = 150 rpm).

The evaluation of the factors of the four kinetic models investigated is presented in Table 5. The plot of (a) ln (*q*_*e*_ − *q*_*t*_) vs. t (b) tqt vs. t (c) *q*_*t*_ vs. *t*^0.5^ (d) *q*_*t*_ vs. ln t is illustrated in Fig 7a–7d. The kinetics of the adsorption of RhB and COD reduction were characterized by the pseudo second order in terms of the closeness of the correlation coefficient to 1, indicating that the kinetics of adsorption of RhB and COD reduction on the acid modified banana peel can better be explained by the pseudo second order model. It was observed from the data that the initial adsorption rate (mins) increases with the increase in the initial adsorbate concentration, suggesting that RhB adsorption and COD reduction were influenced by mass transfer [46]. By implication, the dye molecule was adsorbed by reaching the surface of the adsorbent at a very short equilibrium time. This may be possibly attributed to the active sites and functional groups present on the surface of the adsorbent. Since the kinetics of the uptake capacity of the acid modified banana peel obeyed the pseudo second order model, it is therefore considered that RhB removal and COD reduction were a rate controlling mechanism as a result of the formation of a chemisorptive bond between the adsorbent and the adsorbate [47].

**Table 5:** Kinetic parameters for the adsorption of RhB and COD reduction onto acid modified banana peel at optimum condition.

Parameter	Pseudo first order			Pseudo second order			Elovich				Intraparticle		
	R^2^	K_1_	q_e_(cal) mg/g	R^2^	K_2_	q_e_ (cal) mg/g	R^2^	β	α	q_e_(cal) mg/g	R^2^	K_1_	q_e_(cal) mg/g
RhB	0.8616	0.0295	25.55	0.9994	0.0048	11.4155	0.9399	0.3210	8.4668	18.279	0.9342	0.5077	10.372
COD	0.8852	0.0338	12.49	0.9919	0.0031	14.0905	0.9669	0.3640	1.8112	13.769	0.9607	0.7204	12.281

### Mechanism of adsorption

The mechanism of adsorption was studied to describe the nature of adsorption. This was achieved using the interpretation of the intraparticle diffusion model. Controlling steps that govern the rate of adsorption can be influenced by (1) mass transfer across the external boundary, (2) adsorption at the internal and external surface which depends on the binding process, and (3) diffusion of the adsorbate to the site of adsorption, which could either be through the liquid pores or by solid surface mechanism through pore diffusion. It was observed that the plot of qt vs. *t*^0.5^ was linearized for the uptake of both RhB and COD, although they did not pass through the origin. It could be suggested that the intraparticle diffusion is not the only rate controlling step in the process of adsorption, which implies that the adsorption of RhB and COD reduction onto acid modified banana peel also occurred through boundary layer diffusion.

**Fig 8:**
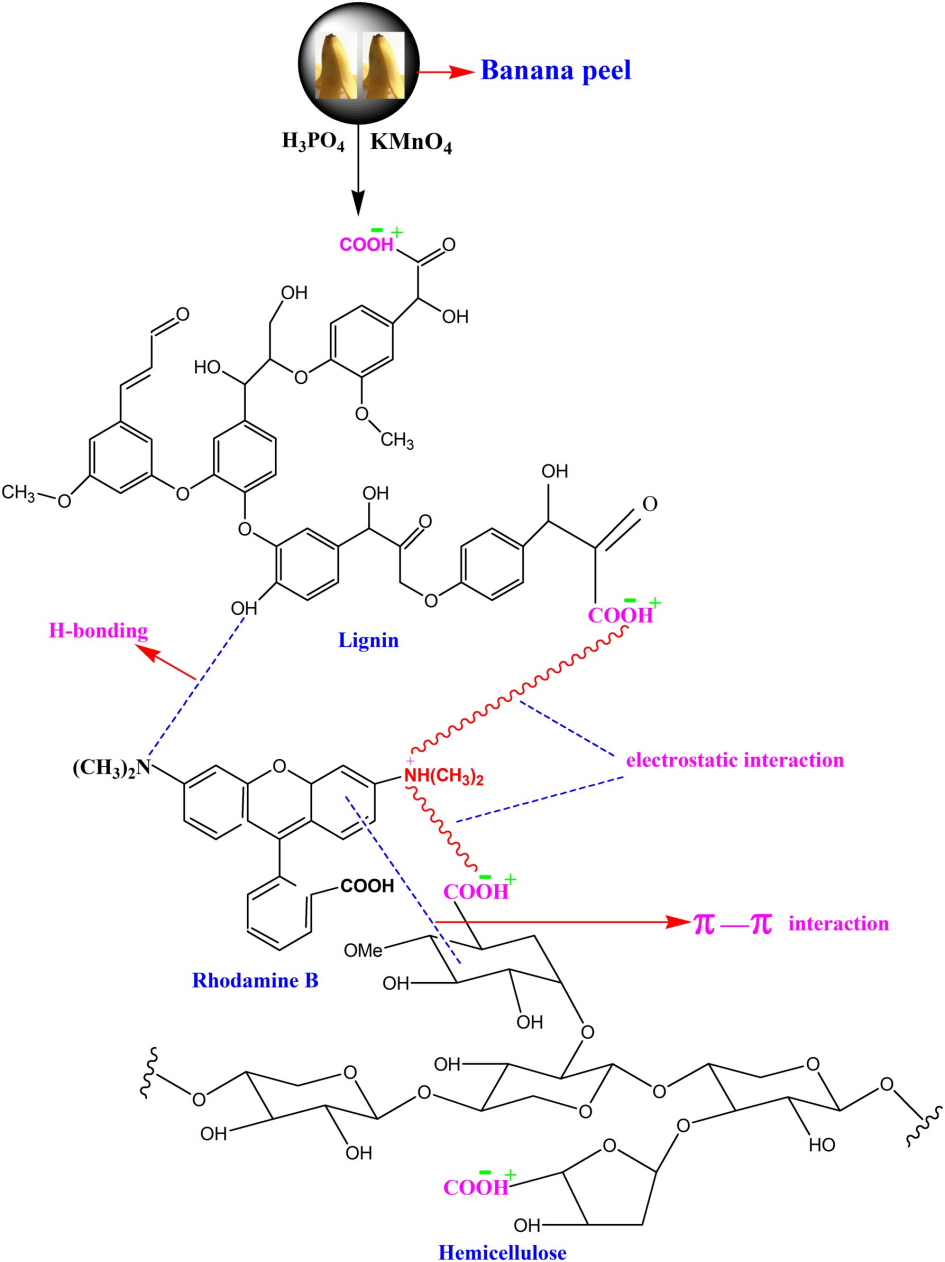
Schematic representation of adsorption mechanism of banana peel and its interaction with RhB.

The banana is the member of Musaceae family and is mainly found in the Malaysian and Indonesian regions of the Asian continent. Banana peels are the waste material of bananas and contain high amounts of hemicelluloses and lignin. These constituents contain various active functional groups that have significant influence on the adsorption or the removal of analytes (dyes, metals, etc.). This is achieved by the interaction or binding which occurs between a solid surface and adsorbate [48]. Investigations revealed that two major factors influenced the adsorption process. The factors are the structure of the adsorbate and the functional groups present on the adsorbent surface. Rhodamine B is a cationic dye with two amino groups and one carboxylic group. According to FTIR spectra, the findings indicated that the acidic functional groups such as the carboxylic groups and the hydroxyl group were available on the sites as a result of the modification of the surface of the peels. These functional groups might be responsible for the uptake of positively charged RhB molecules. Electrostatic attraction, h-bonding and π-π interaction could possibly occur between the negative charged surface and positive charge +NH-CH_3_Rh B molecules (Fig 8) [49].

## Conclusion

The adsorption of RhB and COD reduction using acid modified banana peel was investigated under the operational condition of pH, adsorbent dosage and contact time. The optimized conditions were achieved at pH 2 and 0.2 g/L of adsorbent amount within 60 minutes contact time. The equilibrium adsorption revealed that the experimental data fitted better to the Langmuir isotherm model for the removal of RhB. This suggests that adsorption was on a monolayer on the surface of the adsorbent. Meanwhile, it was observed that the reduction in COD was more suitable on a heterogeneous surface. The kinetic experiments followed the pseudo second order model, which implies adsorption by chemisorption. It was concluded that the intraparticle diffusion was not the only rate controlling step for the adsorption mechanism, since there was influence of adsorption along the boundary surface. The acidic functional groups such as the carboxylic groups and the hydroxyl group available on the surface of banana peel might be responsible for the removal of positively charged RhB molecules.

## Supporting information

S1 Appendix Response output for removal of RhB and COD reduction on acid modified banana peel using a central composite design. (DOCX)
