# Supplementary material for: Adsorption of Rhodamine B dye from aqueous solution onto acid treated banana peel: Response surface methodology, kinetics and isotherm studies
Source: PLoS One. 2019 May 15;14(5):e0216878. doi: 10.1371/journal.pone.0216878 (PMC6519838; doi:10.1371/journal.pone.0216878)
Supplement: S1 Appendix — (DOCX) [file pone.0216878.s001.docx]

**S1 Appendix 1.** Response output for removal of RhB and COD reduction on acid modified banana peel using a central composite design.

| **Run** | $\boldsymbol{x}_{\boldsymbol{1}}$ | $\boldsymbol{x}_{\boldsymbol{2}}$ | $\boldsymbol{x}_{\boldsymbol{3}}$ | **Modified banana peel** | | | |
| --- | --- | --- | --- | --- | --- | --- | --- |
|  |  |  |  | **Percentage removal efficiency** | | | |
|  |  |  |  | **Dye** | | **COD** | |
|  |  |  |  | **Actual** | **Predicted** | **Actual** | **Predicted** |
| 1 | 0.000 | -1.682 | 0.000 | 65.70 | 59.24 | 62.30 | 64.83 |
| 2 | -1.000 | 1.000 | -1.000 | 21.50 | 23.18 | 61.71 | 57.74 |
| 3 | -1.000 | -1.000 | -1.000 | 89.82 | 81.43 | 69.96 | 69.12 |
| 4 | 0.000 | 1.682 | 0.000 | 26.54 | 20.83 | 51.50 | 50.95 |
| 5 | -1.682 | 0.000 | 0.000 | 40.40 | 37.66 | 65.87 | 62.51 |
| 6 | 1.000 | -1.000 | 1.000 | 28.10 | 28.04 | 48.17 | 45.10 |
| 7 | -1.000 | -1.000 | 1.000 | 60.70 | 50.58 | 81.00 | 81.06 |
| 8 | 0.000 | 0.000 | 0.000 | 18.40 | 16.41 | 59.00 | 52.56 |
| 9 | 0.000 | 0.000 | 1.682 | 58.79 | 71.19 | 60.50 | 59.57 |
| 10 | 0.000 | 0.000 | 0.000 | 12.60 | 12.14 | 23.18 | 29.64 |
| 11 | 0.000 | 0.000 | 0.000 | 32.40 | 32.83 | 63.04 | 65.84 |
| 12 | 1.000 | 1.000 | -1.000 | 30.20 | 41.71 | 73.00 | 75.73 |
| 13 | 0.000 | 0.000 | 0.000 | 42.70 | 49.85 | 65.22 | 65.02 |
| 14 | 1.682 | 0.000 | 0.000 | 23.20 | 27.99 | 58.70 | 64.43 |
| 15 | -1.000 | 1.000 | 1.000 | 42.49 | 32.90 | 56.22 | 52.72 |
| 16 | 1.000 | -1.000 | -1.000 | 35.43 | 32.90 | 39.56 | 52.72 |
| 17 | 0.000 | 0.000 | 0.000 | 25.20 | 32.90 | 48.22 | 52.72 |
| 18 | 0.000 | 0.000 | -1.682 | 23.70 | 32.90 | 55.87 | 52.72 |
| 19 | 0.000 | 0.000 | 0.000 | 48.91 | 32.90 | 70.22 | 52.72 |
| 20 | 1.000 | 1.000 | 1.000 | 23.70 | 32.90 | 47.20 | 52.72 |
